# Ultrasonographic features of IgG4-related kidney disease: diagnostic value and correlation with clinical-pathological findings

**DOI:** 10.3389/fmed.2025.1664308

**Published:** 2025-12-17

**Authors:** Boxiong Wei, Yi Liu, Suxia Wang, Yuhong Shao, Xiuming Sun, Luzeng Chen, Xiumei Zhang

**Affiliations:** 1 Department of Ultrasound, Peking University First Hospital, Beijing, China; 2 Department of Urology, Peking University First Hospital, Beijing, China; 3 Laboratory of Electron Microscopy, Pathological Center, Peking University First Hospital, Beijing, China

**Keywords:** IgG4-related kidney disease, ultrasonography, tubulointerstitial nephritis, treatment monitoring, disease activity

## Abstract

**Objective:**

To systematically characterize the ultrasonographic features of biopsy-proven IgG4-related kidney disease (IgG4-RKD) and to evaluate their correlation with key clinicopathological parameters.

**Methods:**

In this retrospective study, the ultrasonographic, clinical, laboratory, and pathological data of 15 patients with biopsy-confirmed IgG4-RKD were analyzed. Key sonographic features, including renal size, parenchymal echotexture, focal lesions, and resistive index, were evaluated and correlated with clinicopathological findings.

**Results:**

The cohort consisted of predominantly older males (86.7%) with frequent multi-organ involvement (60.0%). The most common ultrasonographic pattern included bilateral involvement (93.3%), increased or heterogeneous echogenicity (73.3%/60.0%), multiple hypoechoic areas (46.7%), and enlargement of at least one kidney (>12 cm) in 40.0% of patients. Increased renal length showed a significant positive correlation with serum IgG4 levels (*r* = 0.63, 95% confidence interval (CI) [0.17, 0.86], *p* < 0.05), and parenchymal thickness showed a significant negative correlation with serum C3 levels (*r* = −0.58, 95% CI [−0.84, −0.10], *p* < 0.05). Furthermore, parenchymal thickness was moderately correlated with serum creatinine (*r* = 0.51, *n* = 15, 95% CI [0.00, 0.81], p < 0.05). Ultrasound also effectively monitored therapeutic response, with 83.3% of followed patients showing structural improvement.

**Conclusion:**

Ultrasonography in IgG4-RKD reveals a characteristic pattern of findings that not only serve a descriptive role but also quantitatively correlate with serological disease activity and renal dysfunction. Therefore, ultrasound is a valuable, non-invasive tool for initial assessment, guiding diagnosis, and monitoring therapeutic response in the clinical management of IgG4-RKD.

## Introduction

Chronic kidney disease represents a major global public health challenge, and identifying the specific characteristics of its diverse underlying causes is crucial for improving diagnosis and management (1). One such systemic cause that is increasingly recognized is IgG4-related disease (IgG4-RD), an inflammatory condition with characteristic tumefactive lesions and lymphoplasmacytic infiltrates containing abundant IgG4-positive plasma cells (2, 3). First recognized in 2003, this disease can affect virtually any organ system (4). The condition typically develops in middle-aged to elderly patients, showing male predominance in most organ manifestations (5).

Renal involvement, known as IgG4-related kidney disease (IgG4-RKD), develops in approximately 30% of IgG4-RKD patients (6). The most common renal manifestation is tubulointerstitial nephritis, although membranous glomerulonephritis may also occur (7). Recent data from 125 biopsy-proven cases show that 94% of patients develop tubulointerstitial nephritis, while 16% have concurrent glomerular disease (7).

Current imaging relies mainly on CT and MRI for diagnosing IgG4-RKD. CT typically shows bilateral kidney enlargement with multiple, peripheral, or rim-like low-density lesions (6). MRI reveals bilateral, multiple renal nodules with specific signal patterns (8). Studies using diffusion-weighted MRI report better lesion detection rates (100%) compared to standard T2-weighted sequences (77.4%) (8). However, these methods require contrast agents, involve radiation exposure, and incur substantial costs.

Ultrasound evaluation of IgG4-RKD has received limited attention. Existing literature mainly consists of isolated case reports (9). Some studies mention that ultrasound may show kidney swelling or hypoechoic areas (10), but systematic ultrasound assessment is lacking.

Ultrasound has several practical advantages: it provides real-time imaging, avoids radiation, allows repeated examinations, costs less than other modalities, and is widely available. Given that kidney involvement is common in IgG4-RD and requires long-term follow-up, standardized ultrasound protocols could improve patient care (11). The formal diagnosis of IgG4-RKD itself relies on a comprehensive integration of clinical, serological, imaging, and pathological findings, as outlined in established diagnostic criteria (12). This is noteworthy because while research into other immune-mediated kidney diseases, such as diabetic or membranous nephropathy, has advanced to the level of molecular pathogenesis and targeted biological therapies (13, 14), a systematic characterization of the fundamental imaging features of IgG4-RKD by an accessible modality like ultrasound remains a critical unmet need. This knowledge gap is partly due to the disease’s relatively recent recognition and its rarity, which makes accumulating large cohorts for systematic study challenging. Therefore, this study aims to address this gap by systematically characterizing the ultrasonographic features in biopsy-confirmed IgG4-RKD patients and correlating these findings with their comprehensive clinical and pathological data.

## Materials and methods

### Study design and ethics approval

This retrospective study analyzed the ultrasonographic features of IgG4-related kidney disease (IgG4-RKD) in patients diagnosed between January 2015 and December 2023. The studies involving humans were approved by the Institutional Review Board of Peking University First Hospital, Beijing, China. All procedures were conducted in accordance with institutional and national ethical standards. The requirement for written informed consent was waived due to the retrospective nature of the study.

### Patient selection

We identified 15 patients with biopsy-confirmed IgG4-RKD from the medical records at Peking University First Hospital. Inclusion criteria were: (1) pathologically confirmed IgG4-RKD according to the 2011 and 2020 IgG4-RKD diagnostic criteria (12, 15); (2) availability of complete renal ultrasonographic examination data; and (3) complete clinical and laboratory data. Patients with concurrent kidney diseases that could confound ultrasonographic findings (such as diabetic nephropathy, lupus nephritis, or other primary glomerulonephritis) were excluded. Specifically for lupus nephritis, all patients were screened, and those positive for antinuclear antibodies (ANA) were confirmed to not have systemic lupus erythematosus based on negative anti-dsDNA antibody results and a failure to meet the 2012 Systemic Lupus International Collaborating Clinics classification criteria or the 2019 European League Against Rheumatism/American College of Rheumatology classification criteria for systemic lupus erythematosus.

### Ultrasonographic examination

All patients underwent standardized renal ultrasonography using high-resolution ultrasound systems (GE Logiq 9, GE Logiq E9, Philips HDI 5000, Philips EPIQ 7, Aloka Prosound F75, Esaote Mylab90, Toshiba Aplio 500) with 1–5 MHz convex transducers. Although multiple ultrasound systems were used, all examinations followed standardized image acquisition protocols to minimize inter-device variability. Examinations were performed by experienced sonographers who were blinded to the patients’ clinical information. Both gray-scale and color Doppler imaging were performed in all patients. Gray-scale evaluation included kidney size measurements, parenchymal thickness assessment, echogenicity and corticomedullary differentiation evaluation, and documentation of focal lesions. Doppler evaluation with resistive index (RI) measurement in interlobar arteries was available in 66.7% (10/15) of patients.

### Ultrasonographic evaluation

Ultrasonographic images were retrospectively reviewed by two ultrasound radiologists (with 8 and 10 years of experience, respectively), who were blinded to clinical outcomes. Discrepancies were resolved by consensus. The following parameters were evaluated:

Renal size and morphology: Kidney length was categorized as enlarged (>12 cm), normal (9–12 cm), or small (<9 cm) (16).

Parenchymal characteristics: Parenchymal thickness, echogenicity (normal, increased, or heterogeneous), and corticomedullary differentiation (preserved or impaired).

Focal lesions: Presence of hypoechoic/anechoic areas or hyperechoic foci.

Associated findings: Presence of hydronephrosis, calculi, or simple cysts.

Hemodynamic parameters: Renal arterial resistive index (RI), with values >0.7 considered elevated (17).

### Histopathological analysis

The diagnosis for all patients was pathologically confirmed using formalin-fixed, paraffin-embedded tissue obtained from percutaneous core needle biopsies. No surgical nephrectomy specimens were included in this study.

For light microscopy, 3-μm sections were stained with Hematoxylin and Eosin, Periodic acid–Schiff, Masson’s trichrome, and Jones methenamine silver. For immunofluorescence, 3-μm cryostat sections were stained with polyclonal fluorescein isothiocyanate-conjugated antibodies to IgG, IgM, IgA, C3, C1q, and fibrinogen.

Immunohistochemical staining was performed on 3-μm paraffin-embedded sections. The staining was performed manually. After antigen retrieval by pronase digestion, the sections were blocked with peroxidase-blocking buffer (Zhongshan Golden Bridge, ZSGB-BIO, China) for 20 min at room temperature and 3% BSA for 30 min at 37 °C, and then incubated with polyclonal rabbit anti-human IgG (1:100 dilution, Dako, F0202) and IgG4 (1:500 dilution, Dako, A0136). Secondary antibodies were applied and detection was performed using a polymer-based detection system (Zhongshan Golden Bridge, ZSGB-BIO, China) with diaminobenzidine as the chromogen. Random visual fields were acquired on a DM2500 light macroscope (Leica, Wetzlar, Germany). The diagnostic criteria for IgG4-RKD on histopathology included the presence of >10 IgG4-positive plasma cells per high-power field (HPF) and a ratio of IgG4-positive to IgG-positive plasma cells >40%.

For electron microscopy, specimens were fixed in 2.5% glutaraldehyde, postfixed in 1% osmium tetroxide, and embedded in Epon 812 resin. Ultrathin sections were stained with uranyl acetate and lead citrate and examined by a transmission electron microscope (JEM-1400 flash, Jeol, Tokyo, Japan).

### Statistical analysis

All statistical analyses were performed using SPSS software (version 26.0, IBM, Armonk, NY). Continuous variables were assessed for normality and presented as mean ± standard deviation (SD) for normally distributed data, or as median (range) for non-normally distributed data. For key laboratory parameters such as serum creatinine and IgG4, both metrics were reported to fully characterize their distribution. Categorical variables were presented as percentages. All percentages were calculated based on the number of patients with available data for each specific parameter.

Correlations between ultrasonographic features and clinical/laboratory parameters were analyzed using Fisher’s exact test for categorical variables and Student’s *t*-test or Mann–Whitney *U* test for continuous variables. Correlations between two continuous variables, such as sonographic measurements and laboratory parameters, were assessed using the Pearson or Spearman rank correlation coefficient. The concordance between specific sonographic features and histopathological findings was evaluated using cross-tabulation, and sensitivity was calculated to assess the ability of an ultrasound sign to detect a specific pathology within the patient cohort.

A two-tailed *p*-value of < 0.05 was considered statistically significant.

## Results

### Patient demographics and disease characteristics

The study included 15 patients with biopsy-confirmed IgG4-RKD, comprising 13 males (86.7%) and 2 females (13.3%). The mean age at diagnosis was 63.7 ± 7.9 years (range: 48–80 years). The median duration of disease prior to diagnosis was 11 months (range: 1 week to 11 years). Among these patients, 33.3% (5/15) had a disease duration of 6 months or less, 40.0% (6/15) had a duration between 6 months and 2 years, and 26.7% (4/15) had a duration greater than 2 years.

### Clinical presentation and disease onset

The initial clinical manifestations that led to medical attention varied among patients. Elevated serum creatinine was detected incidentally in 60.0% (9/15) of patients during routine examinations or evaluations for other conditions. Symptomatic urinary abnormalities, including proteinuria and increased urinary foam, were the primary complaint in 20.0% (3/15) of patients. Additional presentations included poor appetite (13.3%, 2/15) and polyuria with weight loss (6.7%, 1/15).

Two patterns of disease onset were observed. In 26.7% (4/15) of patients, renal manifestations were the initial presentation of IgG4-related disease. In the majority of cases (73.3%, 11/15), renal involvement was diagnosed subsequently, following the identification of IgG4-related disease in other organs.

### Multi-organ involvement

Multi-organ involvement was a common feature, with 60.0% (9/15) of patients having three or more organs affected. Specifically, 20.0% (3/15) had isolated renal disease, 20.0% (3/15) had two organs involved, and 46.7% (7/15) had extensive disease affecting five or more organs. Beyond the kidneys (100%), the most frequently involved organs were lymph nodes (60.0%), lungs (46.7%), pancreas (26.7%), and bile ducts (26.7%).

### Laboratory findings

As shown in Table 1, laboratory evaluation revealed several key features. Impaired renal function was common, with 86.7% of patients presenting with elevated serum creatinine (median: 218.0 μmol/L).

**Table 1:** Laboratory features of patients with IgG4-RKD (*n* = 15).

Parameter	Value
Renal function	
Serum creatinine (μmol/L), mean ± SD	237.9 ± 113.5
Serum creatinine (μmol/L), median (range)	218.0 (119.5–470)
Elevated creatinine (>133 μmol/L), % (*n*/*N*)	86.7% (13/15)
Immunological markers	
IgG4 (g/L), mean ± SD	2.54 ± 1.49
IgG4 (g/L), median (range)	2.80 (1.36–4.92)
IgG4 elevation (>1.35 g/L), % (*n*/*N*)	100% (15/15)
Elevated IgG4/IgG ratio (>40%), % (*n*/*N*)	42.9% (3/7)
Complement levels	
Decreased C3, % (*n*/*N*)	100% (11/11)
Decreased C4, % (*n*/*N*)	81.8% (9/11)
Urinary findings	
Proteinuria (any degree), % (*n*/*N*)	100% (15/15)
Mild proteinuria (+ or trace), % (*n*/*N*)	80.0% (12/15)
Moderate to severe proteinuria (≥2+), % (*n*/*N*)	20.0% (3/15)
Other markers	
ANA positivity, % (*n*/*N*)	33.3% (2/6)
Elevated ESR, % (*n*/*N*)	100% (5/5)
Elevated CRP, % (*n*/*N*)	85.7% (6/7)
Elevated IgE, % (*n*/*N*)	71.4% (5/7)

ANA, antinuclear antibody; CRP, C-reactive protein; ESR, erythrocyte sedimentation rate; IgE, immunoglobulin E; IgG4, immunoglobulin G4; SD, standard deviation. Normal reference ranges at our institution are as follows: C3, 0.6–1.5 g/L; C4, 0.12–0.36 g/L; ESR, 0–15 mm/h for males and 0–20 mm/h for females; CRP, 0–8 mg/L; Total IgE, <100 kU/L. ANA was considered positive at a titer of ≥1:100. Denominators vary due to differences in data availability for each test.

The defining serological characteristic was markedly elevated serum IgG4, found in all patients (100%), with a mean of 2.54 ± 1.49 g/L (median: 2.80; range: 1.36–4.92 g/L). Hypocomplementemia was also common, with C3 and C4 levels reduced in 100 and 81.8% of tested patients, respectively. Other notable immunological and inflammatory markers included an elevated IgG4/IgG ratio (in 42.9% of those tested), ANA positivity (33.3%), elevated IgE (71.4%), and high levels of ESR and CRP.

### Pathological features

**Figure 1:**
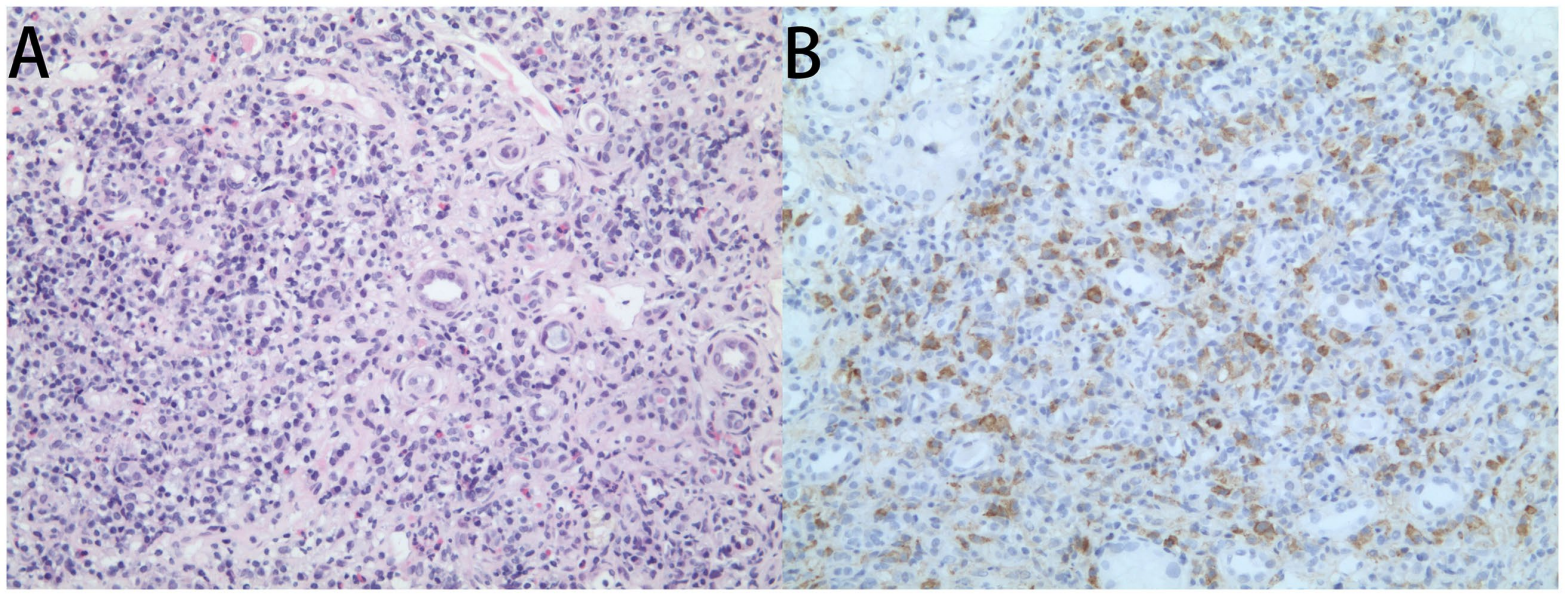
Representative histopathological findings in IgG4-related kidney disease. **(A)** Hematoxylin and Eosin staining showing dense interstitial infiltration of lymphocytes and plasma cells. **(B)** Immunohistochemical staining for IgG4 showing numerous IgG4-positive plasma cells (stained brown) within the interstitium.

Pathological analysis of renal biopsies demonstrated characteristic histopathological features of IgG4-RKD. Glomerular structures were predominantly preserved, with 66.7% (10/15) of biopsies showing no significant glomerular abnormalities, while ischemic sclerosis was seen in 13.3%. In contrast, widespread tubular changes were present in 86.7% (13/15) of biopsies, including tubular atrophy, degeneration, lysosomal accumulation in the tubular epithelium (53.3%), and microvilli detachment (33.3%). The most prominent changes were interstitial; a dense inflammatory cell infiltration was observed in 86.7% of biopsies, composed of lymphocytes (86.7%), plasma cells (80.0%), and fewer eosinophils (26.7%). Interstitial fibrosis, frequently displaying the characteristic “storiform” pattern, was identified in 73.3% of biopsies (Figure 1A). Immunohistochemical staining for IgG4 was performed on seven patients (46.7% of the cohort) (Figure 1B). All seven of these patients showed a dense infiltration of IgG4-positive plasma cells (>10/HPF). However, only three of these seven patients also met the criterion for an elevated IgG4/IgG ratio (>40%). Electron microscopy findings were supportive of the diagnosis, revealing interstitial infiltration by plasma cells and the characteristic finding of sparse, granular electron-dense deposits within the tubular basement membranes.

### Ultrasonographic findings

The ultrasonographic findings for the 15 patients are detailed in Table 2. The predominant pattern was bilateral renal involvement, observed in 93.3% (14/15) of patients, with the majority also having well-preserved renal contours (93.3%).

**Table 2:** Ultrasonographic characteristics of kidneys in IgG4-RKD (*n* = 15).

Parameter	Right kidney	Left kidney	Combined
Size and morphology			
Length (cm), mean ± SD	11.8 ± 1.0	11.6 ± 0.9	
Length >12 cm, % (*n*/*N*)	20.0% (3/15)	33.3% (5/15)	
Length 9–12 cm, % (*n*/*N*)	80.0% (12/15)	66.7%(10/15)	
Length <9 cm, % (n/N)	0% (0/15)	0% (0/15)	
Clear contour, % (*n*/*N*)	93.3% (14/15)	93.3% (14/15)	
Parenchymal features			
Thickness (cm), mean ± SD	1.7 ± 0.3	1.6 ± 0.3	
Echogenicity patterns, % (*n*/*N*)			
Increased echogenicity			73.3% (11/15)
Heterogeneous echogenicity			60.0% (9/15)
Impaired corticomedullary differentiation			60.0% (9/15)
Hypoechoic/anechoic areas			46.7% (7/15)
Punctate hyperechoic foci			33.3% (5/15)
Associated findings, % (*n*/*N*)			
Hydronephrosis	0% (0/15)	0% (0/15)	0% (0/15)
Renal calculi	13.3% (2/15)	6.7% (1/15)	20.0% (3/15)
Simple cysts	20.0% (3/15)	13.3% (2/15)	26.7% (4/15)
Hemodynamic parameters			
RI value, mean ± SD (*n* = 10)			0.67 ± 0.08
RI > 0.7, % (*n*/*N*)			40.0% (4/10)

RI, resistive index; SD, standard deviation. Echogenicity patterns and hemodynamic parameters were evaluated and reported as combined findings for both kidneys.

While the mean renal length was within the upper limits of normal at 11.8 ± 1.0 cm for the right kidney and 11.6 ± 0.9 cm for the left, further analysis revealed that 20.0% (3/15) of right kidneys and 33.3% (5/15) of left kidneys measured >12 cm in length. Overall, 40.0% of patients (6/15) had at least one enlarged kidney (>12 cm). No atrophic kidneys (<9 cm) were found in the cohort.

The most characteristic parenchymal abnormalities were increased echogenicity, present in 73.3% (11/15) of patients, and a heterogeneous echotexture, seen in 60.0% (9/15). Impaired corticomedullary differentiation was also common, observed in 60.0% of cases. Furthermore, multiple focal hypoechoic or anechoic areas were a notable finding in 46.7% (7/15) of patients, while punctate hyperechoic foci were identified in 33.3% (5/15).

Doppler analysis was available for 10 patients (66.7%), revealing a mean resistive index (RI) of 0.67 ± 0.08 (range: 0.56–0.81). An elevated RI (>0.7) was present in 40.0% (4/10) of these patients. The distribution of RI values was as follows: 20.0% were between 0.5–0.6, 40.0% between 0.61–0.7, 30.0% between 0.71–0.8, and 10.0% were >0.8.

**Figure 2:**
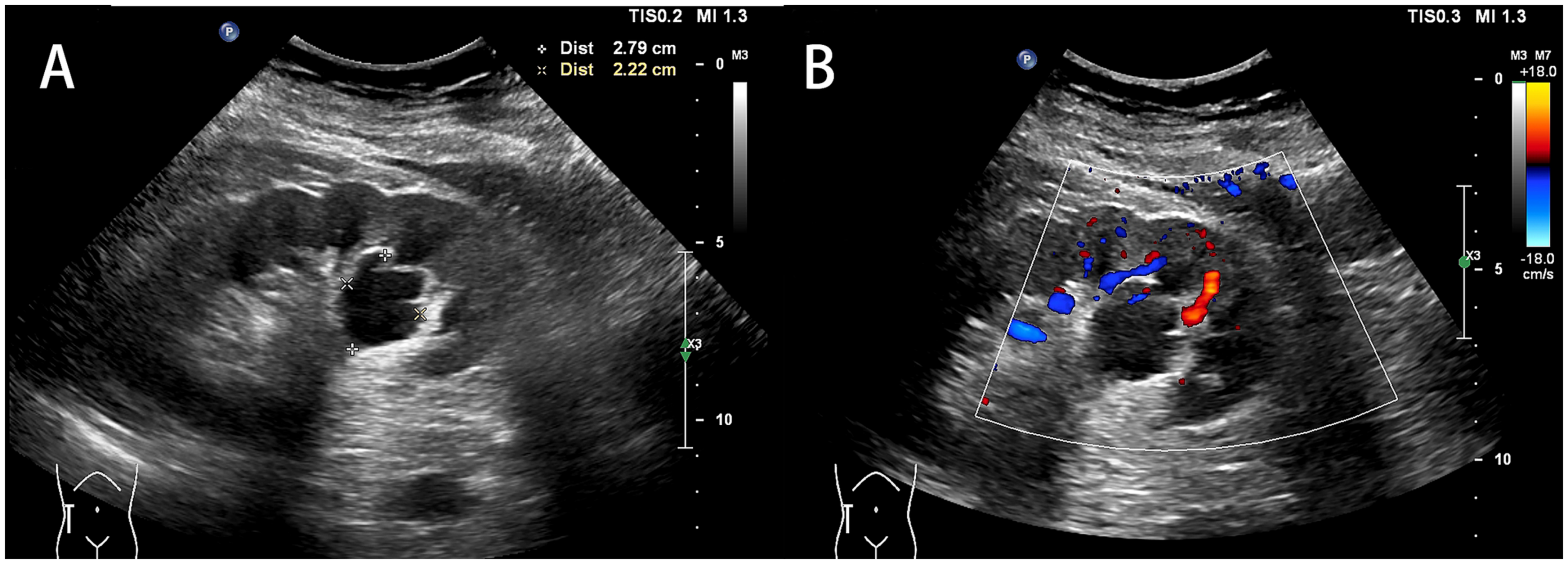
Ultrasonographic features of IgG4-related kidney disease mimicking renal cell carcinoma. **(A)** Grayscale (2D) ultrasonography reveals a relatively well-defined, heterogeneous, hypoechoic mass-like lesion in the kidney, distorting the normal renal architecture. **(B)** Color Doppler flow imaging shows some peripheral blood flow around the mass, but no definite vascular signals are detected within the lesion itself.

Notably, one patient (6.7%) presented with a right renal focal mass-like lesion that was initially misdiagnosed as renal cell carcinoma, appearing as a heterogeneous, hypoechoic mass on grayscale imaging (Figure 2A) with only peripheral vascularity on color Doppler (Figure 2B). Other associated findings were also documented; hydronephrosis was absent in all cases, while renal calculi and simple cysts were detected in 20.0 and 26.7% of patients, respectively.

### Combination of sonographic features

While individual ultrasonographic abnormalities were frequent, the combination of features provided a more consistent pattern in identifying patients with IgG4-RKD. The presence of at least two of the primary abnormalities (renal enlargement, increased/heterogeneous echogenicity, hypoechoic areas, or an elevated RI) was observed in a large majority of the cohort (86.7%). Furthermore, a combination of at least three of these key features was found in 53.3% of patients.

Specific combinations were also analyzed. A pattern of renal enlargement combined with increased echogenicity was present in 60.0% of patients, while a combination of heterogeneous echotexture and multiple hypoechoic areas was seen in 40.0%. The triad of renal enlargement, heterogeneous echogenicity, and an elevated RI was observed in 26.7% of patients.

### Correlation analyses

#### Correlation of sonographic features with laboratory markers and systemic disease burden

Sonographic measurements demonstrated significant correlations with key laboratory markers. Specifically, increased renal length showed a moderate positive correlation with serum IgG4 levels (*r* = 0.63, 95% confidence interval (CI) [0.17, 0.86], *p* < 0.05); for instance, patients with kidney length >12 cm had a higher mean IgG4 level than those with smaller kidneys (3.42 g/L vs. 1.97 g/L). Similarly, parenchymal thickness was moderately correlated with serum creatinine (*r* = 0.51, 95% CI [0.00, 0.81], *p* < 0.05), a finding underscored by the observation that all patients (7/7) with thickness >1.8 cm also had creatinine levels >200 μmol/L, compared to only 37.5% of those with thinner parenchyma.

Furthermore, parenchymal changes were linked to complement levels. Parenchymal thickness was inversely correlated with serum C3 levels (*r* = −0.58, *n* = 15 95% CI [−0.84, −0.10], *p* < 0.05), and patients with low C3 universally demonstrated both increased parenchymal thickness and altered echogenicity. The extent of systemic disease burden was also strongly associated with sonographic findings; patients with extensive organ involvement (≥5 organs) were significantly more likely to exhibit renal enlargement (p < 0.05), heterogeneous echogenicity (p < 0.05), and focal hypoechoic areas (*p* < 0.01) compared to those with single-organ disease.

### Sonographic-pathological correlation

Key ultrasonographic features showed high concordance with specific histopathological changes. Heterogeneous echogenicity was a sensitive indicator of underlying interstitial fibrosis (90.9% sensitivity, 84.6% concordance). The presence of multiple hypoechoic areas corresponded well with focal inflammatory infiltrates (83.3% sensitivity, 76.9% concordance). An elevated RI (>0.7) was suggestive of microvascular pathology (75.0% sensitivity, 70.0% concordance), while punctate hyperechoic foci were associated with calcifications or crystal deposits.

### Treatment regimens and response

Twelve of the 15 patients were treated. Glucocorticoids were the primary treatment in all treated patients (100%, 12/12), predominantly as prednisone/prednisolone (91.7%, 11/12) with methylprednisolone used in one case (8.3%, 1/12). Most received glucocorticoid monotherapy (75.0%, 9/12), while some had combination therapy with immunosuppressants (16.7%, 2/12) or immunomodulators (8.3%, 1/12).

Treatment response was favorable in all followed patients (100%, 10/10), with significant improvement in 60.0% (6/10) and partial improvement in 40.0% (4/10). Serum creatinine decreased in all cases. Follow-up ultrasonography, performed at a median of 6 months (range: 3–12 months) after treatment initiation and available for 6 of the 10 treated patients, showed kidney size reduction in 83.3% (5/6) and improved echogenicity in 66.7% (4/6). Hypoechoic areas demonstrated the most rapid response, with resolution in 75.0% (3/4) of relevant cases.

Baseline ultrasonographic features correlated with treatment outcomes, with better functional recovery in patients showing milder initial echogenicity changes.

## Discussion

This study systematically characterized the ultrasonographic features of IgG4-RKD and revealed strong correlations between sonographic findings and clinicopathological markers of disease activity. Our findings confirm the established profile of IgG4-RKD as a systemic disease predominantly affecting older males, with universally elevated serum IgG4 and frequent hypocomplementemia providing a crucial diagnostic context for the imaging results (12, 18). A key finding of this work is demonstrating that sonographic abnormalities are not merely descriptive but quantitatively mirror the systemic disease burden and renal dysfunction, as evidenced by the strong correlations between renal length, parenchymal thickness, and key serological markers.

The sonographic results provided a non-invasive window into the underlying pathology. For instance, heterogeneous echogenicity was a sensitive indicator of interstitial fibrosis, while multiple hypoechoic areas corresponded well with the focal inflammatory infiltrates seen on biopsy (6, 19). This connection is crucial for understanding treatment response, as the resolution of these features on follow-up ultrasound suggests a reduction in active and chronic disease components (20).

Another important finding was the case of a “pseudotumor” presentation initially misdiagnosed as renal cell carcinoma. This recognized diagnostic challenge, further complicated by recent reports of atypical cystic masses and concurrent malignancies like lymphoma (21, 22), underscores the indispensable role of histopathological confirmation when imaging findings are equivocal (23, 24). While the primary finding in our cohort was tubulointerstitial nephritis, the full pathological spectrum is increasingly recognized as complex, potentially including diverse glomerular involvement that can challenge conventional subclassifications (25).

Although the individual sonographic features of IgG4-RKD are not specific, ultrasound offers clear and important value in the clinical management pathway. As a first-line screening tool, its findings can raise clinical suspicion for IgG4-RKD and help speed up the diagnosis (26). Ultrasound also provides essential real-time guidance for renal biopsies, which are often needed for a final diagnosis; this was a role it played in 86.7% of the cases in this study (27). Finally, this study confirms the value of ultrasound for monitoring treatment response. The results show that sonographic changes correlate strongly with serological markers, making it a useful tool for non-invasively tracking disease activity and the effectiveness of treatment (28).

A critical aspect of ultrasound’s utility lies in differentiating IgG4-RKD from its sonographic mimics. For instance, while renal lymphoma can also cause multiple hypoechoic masses, these are typically more well-defined and less vascular compared to the often ill-defined inflammatory areas in IgG4-RKD (29). Other inflammatory conditions, such as lupus nephritis or acute pyelonephritis, may also cause renal enlargement and altered echogenicity. However, these diseases typically lack the multiple, distinct hypoechoic areas characteristic of IgG4-RKD, and instead present with more diffuse parenchymal changes or acute clinical signs of infection (30). This analysis underscores that while no single feature is pathognomonic, evaluating the combined sonographic pattern in conjunction with the clinical context is essential for an accurate differential diagnosis.

The pathogenesis of IgG4-RKD is complex, and our sonographic findings of structural damage are likely the ultimate manifestation of several interconnected molecular pathways.

Recent evidence increasingly points to a “gut-kidney axis,” where an imbalance of gut bacteria (microbial dysbiosis) can trigger systemic inflammation and oxidative stress, thereby promoting renal injury (31–33). This persistent inflammatory state is a key factor, as it can subsequently induce the local activation of the intrarenal renin-angiotensin system (RAS). The activation of RAS, in turn, is a well-established driver of the tubulointerstitial fibrosis—a form of tissue scarring—that was a prominent pathological feature in our cohort (34).

This cascade of events provides a compelling molecular basis for our ultrasonographic observations. The interstitial fibrosis driven by RAS likely contributes directly to the increased and heterogeneous parenchymal echotexture we recorded. Similarly, the inflammation and cellular infiltration are the primary causes of the increased parenchymal thickness. This perspective highlights the value of ultrasound not just as a diagnostic tool, but as a crucial, non-invasive window to visualize the cumulative structural consequences of these complex molecular events.

Baseline ultrasonographic features predicted treatment outcomes: patients with echogenicity changes in <50% of renal parenchyma showed better treatment response than those with extensive involvement (85.7% vs. 33.3%, *p* < 0.05) (35). Punctate hyperechoic foci indicated poorer functional recovery. During treatment, hypoechoic areas resolved first (2–4 weeks), followed by parenchymal thickness normalization (4–8 weeks), with echogenicity changes resolving last (2–6 months) (12). Early kidney size reduction (>10% within 1 month) predicted sustained remission (20).

The insidious nature of IgG4-RKD was evident in this cohort, as a majority of patients (60.0%) were diagnosed incidentally through laboratory findings rather than specific renal symptoms. For most patients (73.3%), renal involvement was discovered only after an extra-renal diagnosis of IgG4-RD had been established. This highlights the need for proactive renal screening in all patients with a known diagnosis of extra-renal IgG4-RD to enable early detection and intervention (18).

The severity of renal involvement also appeared to mirror the overall systemic disease burden. In this study, key laboratory findings, such as high IgG4 levels and hypocomplementemia, were more severe in patients with extensive multi-organ involvement. The immunological background of IgG4-RD is complex and continues to be elucidated. While our study confirms the typical polyclonal elevation of IgG4, recent reports have described rare but significant associations between IgG4-RKD and monoclonal gammopathy, including both Monoclonal Gammopathy of Undetermined Significance and Monoclonal Gammopathy of Renal Significance (36). While our study did not specifically screen for this, these findings highlight the complex interplay between polyclonal and monoclonal immune responses in some patients and suggest the importance of a comprehensive hematological evaluation. The combination of markedly elevated IgG4 and profound complement consumption is a particularly valuable diagnostic clue, helping to differentiate IgG4-RKD from other forms of tubulointerstitial nephritis (35).

Our study was limited by small sample size (*n* = 15), variability in ultrasonographic technique. Additionally, while formal interobserver agreement statistics were not calculated, all images were reviewed by two experienced radiologists to ensure consensus, which minimized interpretive variability. Limited follow-up imaging (available in only 6/10 patients) restricted our ability to fully characterize sonographic evolution during treatment. Future prospective studies with larger cohorts and standardized protocols are needed to establish more definitive ultrasonographic criteria for IgG4-RKD.

## Conclusion

In conclusion, IgG4-RKD presents with a characteristic constellation of sonographic features. While individually non-specific, this pattern strongly correlates with both systemic disease burden and key serological markers of activity. Furthermore, ultrasound effectively tracks structural changes in response to therapy, distinguishing between active inflammation and chronic fibrosis. These findings validate the role of ultrasonography as an essential, non-invasive tool for the comprehensive clinical management of IgG4-RKD, from initial assessment to long-term follow-up.

## Data Availability

The original contributions presented in the study are included in the article/supplementary material, further inquiries can be directed to the corresponding authors.
